# Health system intervention: back of the envelope to statewide transformation of occupational health care delivery

**DOI:** 10.1186/1748-5908-10-S1-A84

**Published:** 2015-08-20

**Authors:** Thomas Wickizer, Gary Franklin, Deborah Fulton-Kehoe

**Affiliations:** 1 Division of Health Services Management and Policy, College of Public Health, The Ohio State University, 1841 Neil Avenue, Columbus, OH 43210, USA; 2 Department of Environmental and Occupational Health Sciences, University of Washington, Box 359116, Seattle, WA 98109, USA

## 

Washington State has been a national leader in efforts to improve the workers' compensation (WC) health care delivery system. In 2002, the WA Department of Labor and Industries (DLI) initiated a major system intervention to improve quality and outcomes in WC health care. Two Centers of Occupational Health and Education (COHE) were developed as pilot sites to test the intervention, which included physician financial incentives to reward the adoption of occupational health best practices, improved care coordination, use of evidence-based protocols to improve clinical care, and development of patient tracking systems. We conducted a rigorous evaluation to determine whether COHE patients, compared with non-COHE comparison group patients, had reduced work disability and decreased disability and medical expenditures (n = 105,607). Throughout the 8 years of evaluation, the research team worked closely with statewide and local COHE advisory groups to maintain critical support for the initiative. The evaluation showed the COHE was associated (p < 0.001) with: (1) a 30% decrease in the likelihood of long-term (one-year) disability, (2) a reduction of 4 disability days per case, and (3) a decrease of $267 in disability costs per claim (and non-significant reduction in medical costs). These evaluation results, coupled with the strong advisory group support, led to the passage of a state law in March 2011 expanding the COHE on a statewide permanent basis, with over 1,800 physicians now participating in the expanded COHE network. Our research demonstrates the importance of: (1) evaluators working closely with key stakeholder groups to engender critical support for a system intervention and to help overcome political opposition that may arise, (2) conducting rigorous evaluation research to determine the effects of the system intervention, and (3) where possible reducing the administrative burden of physicians.

Funding for the COHE system.

